# CCR5Δ32 Genotype Leads to a Th2 Type Directed Immune Response in ESRD Patients

**DOI:** 10.1371/journal.pone.0031257

**Published:** 2012-02-13

**Authors:** Friso L. H. Muntinghe, Wayel H. Abdulahad, Minke G. Huitema, Jeffrey Damman, Marc A. Seelen, Simon P. M. Lems, Bouke G. Hepkema, Gerjan Navis, Johanna Westra

**Affiliations:** 1 Internal Medicine, Vasculair Medicine, University Medical Center Groningen, Groningen, The Netherlands; 2 Rheumatology and Clinical Immunology, University Medical Center Groningen, Groningen, The Netherlands; 3 Internal Medicine, Nephrology, University Medical Center Groningen, Groningen, The Netherlands; 4 Laboratory Medicine, Transplantation Immunology, University Medical Center Groningen, Groningen, The Netherlands; Carl-Gustav Carus Technical University-Dresden, Germany

## Abstract

**Background:**

In patients with end stage renal disease (ESRD) we observed protection from inflammation-associated mortality in CCR5Δ32 carriers, leading to CCR5 deficiency, suggesting impact of CCR5Δ32 on inflammatory processes. Animal studies have shown that CCR5 deficiency is associated with a more pronounced Th2 type immune response, suggesting that in human CCR5Δ32 carriers the immune response may be more Th2 type directed. So, in the present study we determined the Th1-Th2 type directed immune response in ESRD patients carrying and not carrying the CCR5Δ32 genetic variant after stimulation.

**Methodology/Principal Findings:**

We tested this hypothesis by determining the levels of IFN-γ and IL-4 and the distribution of Th1, Th2 and Th17 directed circulating CD4+ and CD8+ T cells and regulatory T cells (Tregs) after stimulation in ESRD patients with (n = 10) and without (n = 9) the CCR5Δ32 genotype. The extracellular levels of IFN-γ and IL-4 did not differ between CCR5Δ32 carriers and non carriers. However, based on their intracellular cytokine profile the percentages IL-4 secreting CD4+ and CD8+ T cells carrying the CCR5Δ32 genotype were significantly increased (p = 0.02, respectively p = 0.02) compared to non carriers, indicating a more Th2 type directed response. Based on their intracellular cytokine profile the percentages IFN-γ and IL-17 secreting T cells did not differ between carriers and non-carriers nor did the percentage Tregs, indicating that the Th1, Th17 and T regulatory response was not affected by the CCR5Δ32 genotype.

**Conclusions/Significance:**

This first, functional human study shows a more pronounced Th2 type immune response in CCR5Δ32 carriers compared to non carriers. These differences may be involved in the previously observed protection from inflammation-associated mortality in ESRD patients carrying CCR5Δ32.

## Introduction

Genetic variability in the chemokine cascades could potentially influence disease outcomes by modifying inflammatory processes. CC-chemokine receptor 5 (CCR5) is one of the chemokine receptors. It is expressed on T cells and monocytes and it is important for recruitment [1], [2]. Several polymorphisms have been described for CCR5. The CCR5Δ32 genetic variant is located on the chromosome 3p21 and consists of a 32-basepair deletion in the open reading frame. It effectively results in functional CCR5 deficiency by absence of CCR5 membrane expression [3]. We observed protection from inflammation-associated mortality in carriers of the deletion 32 allele in end stage renal disease (ESRD), suggesting impact of CCR5Δ32 on the inflammatory process of atherosclerosis [4]. Also in other human populations, characterized by high cardiovascular risk, the presence of the CCR5Δ32 genotype has been associated with better outcome [5]–[8].

In chronic inflammatory processes like atherosclerosis T cells play an important role. Both CD4+ T cells and to a lesser extent CD8+ T cells are present in atherosclerotic lesions [9]–[12]. CD4+ T helper cells can differentiate into three effector lineages based on their cytokine expression: IFN-γ/TNF-α producers (Th1), IL-4 producers (Th2) and IL-17 producers (Th17) [12], [13]. In addition, a small fraction of CD4+ T cells can develop into cells with a regulatory function (Tregs) that are defined by their co-expression of high levels of surface CD25 and intracellular transcription factor forkhead box P3 (FoxP3). These Tregs have the remarkable ability to suppress the proliferation and effector function of other T cells [9], [12]. As with CD4+ T cells, CD8+ T cells can differentiate to T cytotoxic (Tc)1 or Tc2 cell subsets, secreting predominantly Th1 or Th2 cytokines respectively [13].

Atherosclerotic inflammation is regarded as a (partly) Th1 driven condition [12]. The CCR5 receptor is highly expressed T-lymphocytes, on both CD4+ T and CD8+ T cells [2], [11]. In atherosclerotic mice CCR5 deficiency is associated with a more pronounced Th2 type immune response and less TNF-α and IFN-γ production hereby counteracting the Th1 directed Th1/Th2 disequilibrium of atherosclerotic inflammation [14]–[17].

These data fuel the hypothesis that the immune response in carriers of the CCR5Δ32 genotype is more Th2 type directed. Such differences in response might play a role in the protection against inflammation-associated mortality in ESRD in carriers of the CCR5Δ32 genotype. To test this hypothesis we studied the cell mediated immune responses in peripheral mononuclear cells (PBMCs) in ESRD patients. We first determined the extracellular levels of IFN-γ and IL-4 after stimulation of PBMCs, and second the distribution of Th1, Th2 and Th17 directed circulating CD4+ and CD8+ T cells, based on their intracellular cytokine profile after stimulation, and the percentage of Tregs in ESRD patients with and without the CCR5Δ32 genotype.

## Methods

### Objectives

The objective of the present study was to determine possible differences in cell mediated immune response between ESRD carriers and non carriers of the CCR5Δ32 genotype. To test this hypothesis we first determined the IFN-γ and IL-4 levels after stimulation of peripheral mononuclear cells (PBMCs) and secondly the distribution of Type-1, Type-2 and Type-17 directed circulating CD4+ and CD8+ T cells, based on their intracellular cytokine profile, as well as the frequency of FoxP3+ regulatory T cells.

### Participants

Biosamples and data from twenty patients with ESRD were included in this study. These patients were part of an ESRD cohort from a single kidney transplant centre in the Netherlands (University Medical Center Groningen), in whom data and biosamples were collected prior to kidney transplantation. As part of a larger genotyping project, all patients were genotyped as described below. For the current project we randomly selected five homo- and five heterozygous carriers from the cohort. Ten wild type patients were matched with carriers according to time of inclusion, hereby creating similar preservation conditions.

### Genotyping

The genotypes were determined with a PCR-based allelic discrimination assay using primers (Life Technologies) and allele-specific probes (PE Biosystems) as described previously [18]. Patients were grouped by CCR5 genotype, namely those homozygous for the major allele (non-carriers) and those with 1 or 2 deletion alleles (carriers). Patients with one or two deletion alleles were grouped together, as it has been demonstrated that presence of one deletion allele is sufficient to compromise CCR5 function [3].

### Sample preparation and thawing

Heparinized venous blood was obtained from ESRD-patients who gave their informed consent. PBMCs were separated by conventional Ficoll gradient and frozen in 10% DMSO in FCS and stored in liquid nitrogen. PBMCs were thawed and washed twice with RPMI 1640 media (Cambrex Bio Science, Verviers, Belgium), supplemented with 10% heat inactivated fetal calf serum and 50 µg/mL gentamycin (Gibco, Scotland, UK).

### Determination of extracellular cytokine by ELISA

Thawed PBMCs were cultured in a 5 mL polypropylene tubes (BD Biosciences) at 2,5×10^6^ cells/mL per tube, and stimulated with 40 n*M* phorbol myristate acetate (PMA; Sigma-Aldrich, Steinheim, Germany) and 2 n*M* calcium ionophore (Sigma-Aldrich). Culture supernatants were collected over a period of 24 hours to determine the extracellular levels of IFN-γ, and IL-4 cytokines.

Cytokine levels of IFN-γ and IL-4 were measured by commercial sandwich enzyme linked immunoassay (ELISA) kits (Pelikine Compact, Sanquin, Amsterdam, The Netherlands), according to the instructions of the manufacturer.

### Determination of intracellular cytokine by flow cytometry

The following conjugated antibodies were used in flow cytometry: allophycocyanin (APC)–Cy7–conjugated anti-CD69, peridin-chlorophyll protein (PerCP)–conjugated anti-CD8, phycoerythrin (PE)– Cy7–conjugated anti–IL-4, and Alexa Fluor 700–conjugated anti-IFN-γ (all from Becton & Dickinson, Amsterdam, The Netherlands). Alexa Fluor 488–conjugated anti–IL-17, Alexa Fluor 647-conjugated anti-TNF-α, and eFluor605™–conjugated anti-CD3 were obtained from eBioscience (San Diego, CA). To determine the frequency of T cell subsets by measuring intracellular cytokine production, cells were stimulated for 4 hours with 40 n*M* PMA and 2 n*M* calcium ionophore. Brefeldin A (10 µg/mL) was added to inhibit cytokine release. After stimulation, cells were washed in wash buffer (phosphate buffered saline, 5% fetal bovine serum, 0.1% sodium azide; Merck, Darmstadt, Germany) and stained with eFluor 605-conjugated anti-CD3, PerCP–conjugated anti-CD8 and APC-Cy7–conjugated anti-CD69 for 15 minutes at room temperature. Cells were fixed with 100 µl Reagent A (Caltag, An Der Grab, Austria) for 15 minutes. After washing, the pellet was resuspended in 100 µl permeabilization Reagent B (Caltag) and labeled with PE-Cy7–conjugated anti–IL-4, Alexa Fluor 700–conjugated anti-IFN-γ, Alexa Fluor 647-conjugated anti-TNF-α, Alexa Fluor 488–conjugated anti–IL-17, and APC-Cy7–conjugated anti-CD69 for 30 minutes in the dark. After staining, the cells were washed and immediately analyzed on a FACS-LSRII flow cytometer (Becton Dickinson). Seven-color flow cytometric acquisition was performed using FACSDiva software (Becton Dickinson). For all flow cytometry analyses, data were collected for 2×10^5^ cells and plotted using the Win-List software package (Verity Software House, Topsham, ME). Because stimulation reduces surface expression of CD4 on T cells, CD4 T cells were identified indirectly by gating on CD3+ and CD8− lymphocytes, whereas CD8+ T cells were identified by directly gating on CD3+ and CD8+ lymphocytes. Subsets of activated CD4+ and CD8+ T cells in response to mitogenic stimulation were evaluated by double expression of activation marker CD69 and intracellular cytokine production of IFN-γ (for type-1) or IL-17 (for type-17) or IL-4 (for type-2). The unstimulated samples were used as a guide for setting the linear gates to delineate positive and negative populations.

### Determination of regulatory T cell frequencies

PBMCs were washed with cold PBS (pH 7.2) and incubated with appropriate concentrations of PerCP-conjugated anti-CD4, FITC-conjugated anti-CD3 and PE-conjugated anti-CD25 (all purchased from BD) for 30 min at 4°C in the dark. Cells were then washed with cold PBS, followed by fixation and permeabilization in Fix/Perm buffer (FoxP3 staining kit, eBioscience, Uithoorn, The Netherlands) for 45 min at 4°C. Subsequently, cells were washed with cold permeabilization buffer (FoxP3 staining kit, eBioscience, Uithoorn, The Netherlands). To block nonspecific binding, normal rat serum was added for 10 min, followed by the addition of APC-conjugated rat anti-human FoxP3 (eBioscience, Uithoorn, The Netherlands). After incubation for 30 min at 4°C, the cell suspension was washed twice with cold permeabilization buffer, and immediately analyzed on FACS-Calibur (BD). Data were collected for 2×10^5^ cells and plotted using the Win-List software package (Verity Software House, Topsham, ME). Positively and negatively stained populations were calculated by quadrant dot-plot analysis, determined by the isotype matched control antibodies of irrelevant specificity (obtained from BD and eBioscience).

### Ethics

All patients gave written informed consent and the local medical ethics committee from the University Medical Center Groningen (METc UMC Groningen), the Netherlands, gave their approval.

### Statistical methods

Patients were grouped by CCR5 genotype, namely those homozygous for the major allele (non-carriers) and those with 1 or 2 deletion alleles (carriers). The latter were grouped together, as it has been demonstrated that presence of one deletion allele is sufficient to compromise CCR5 function [3]. Data are presented as the median. The nonparametric Mann-Whitney U test was used to compare data from patients with and without the CCR5Δ32 genotype. Differences were considered statistically significant at 2-sided *p* values less than 0.05.

## Results

### Patients

From the 20 patients who were included in this study 1 stimulation test failed due to the fact that the T cells could not be stimulated. Further statistical analyses were performed on the remaining 19 patients. Baseline characteristics are shown in Table 1. There were no statistically significant differences in baseline characteristics between the 2 genotype groups.

**Table 1:** Baseline characteristics.

	CCR5 wild type(n = 9)	CCR5 deletion 32(n = 10)
Gender; male	4 (44.4)	7 (70.0)
Age (year)	51 (14)	51 (13)
Primary kidney disease		
Renal vascular disease and hypertension	3	3
Diabetes mellitus	1	0
Cystic kidney disease	1	3
Pyelonephritis	1	1
Primary oxalosis	1	0
Unknown	2	3
Dialysis duration (days)	1570 (880)	1005 (734)
Hemodialysis	5 (55.6)	4 (44.4)
Body Mass Index	26 (4.94)	25 (3.21)
Systolic bloodpressure (mmHg)	147 (19)	138 (10)
Diastolic bloodpressure (mmHg)	86 (8)	86 (9)

Data are presented as number (percentage), mean (SD).

### Extracellular cytokines of type-1 and type-2 T cells

**Figure 1:**
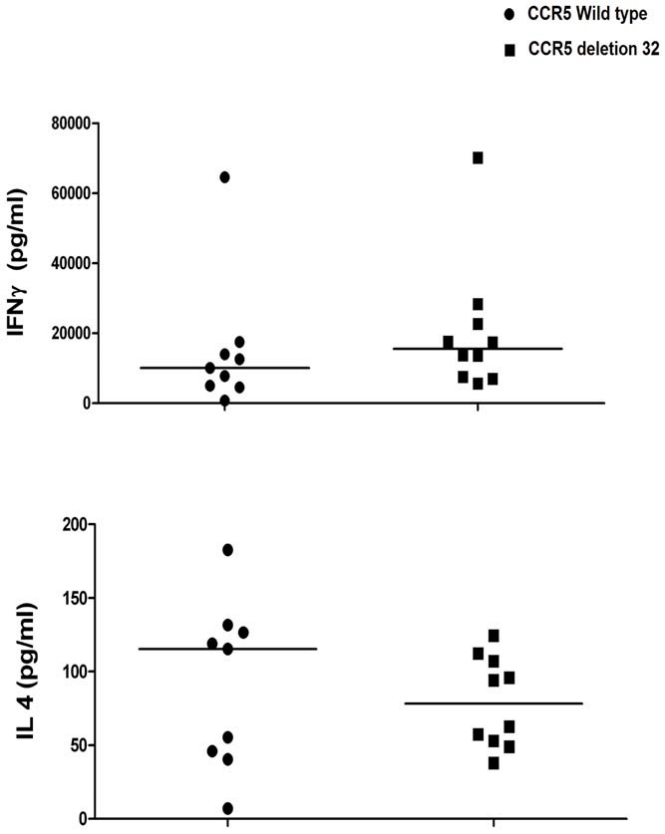
IFN-γ and IL-4 ELISA per 2,5×10^6^ PBMCs in carriers and non carriers of the CCR5Δ32 genotype. Levels of IFN-γ and IL-4 after stimulation from carriers (n = 10) and non carriers (n = 9) are shown. Horizontal lines represent the medians.

To assess the functional capacity of the responding PBMCs the total amount of IFN-γ and IL-4 after stimulation was determined. As shown in Figure 1, the levels of extracellular IFN-γ and IL-4 were not statistically significant different between carriers and non carriers of the CCR5Δ32 genotype.

### Intracellular cytokines of type-1, type-2, and type-17 T cells

**Figure 2:**
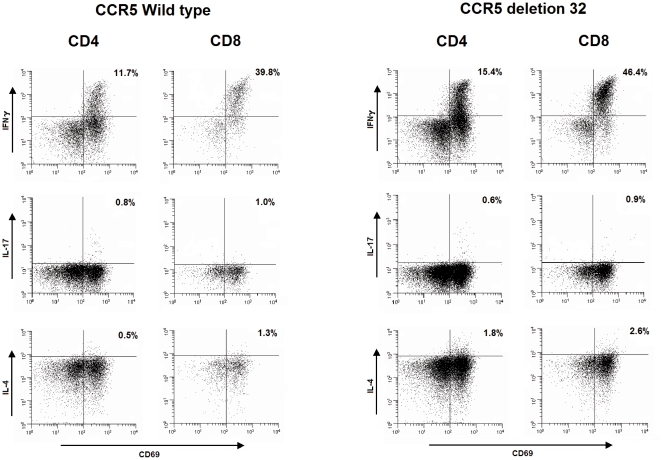
Flow cytometric characterization of CD4 and CD8 T cell subsets from CCR5 wild type (left panel) and CCR5 deletion 32 (right panel). PBMCs were stimulated *in vitro* with PMA and Ca-ionophore for 4 hours in the presence of BFA. The CD4 and CD8 T cell subsets were then assessed for the expression of activation marker CD69 versus intracellular cytokine (IFN-γ, IL-17, and IL-4). The percentage in the upper right corner of each plot represents the net percentage of positive cells.

**Figure 3:**
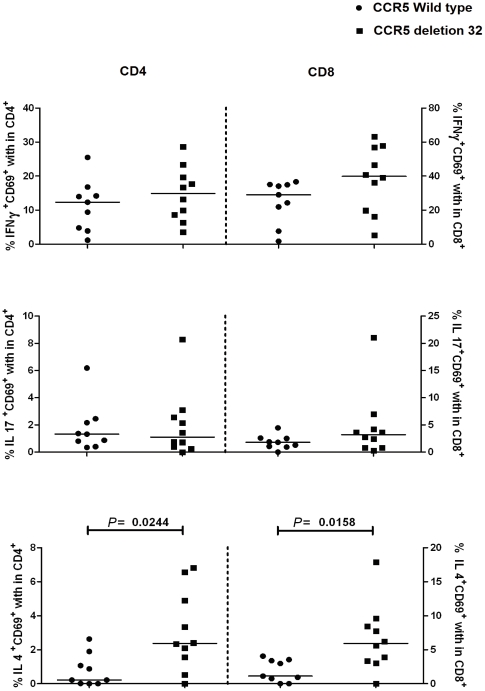
Percentages of IL-4, IL-17 and IFN-γ secreting CD4 and CD8+ T cells in carriers and non carriers of the CCR5Δ32 polymorphism. In the left panel the frequencies of IL-4, IL-17 and IFN-γ secreting cells among CD69+, CD4+ T cells from non carriers (n = 9) and carriers (n = 10) of the CCR5Δ32 genotype are shown. In the right panel the frequencies of IL-4, IL-17 and IFN-γ secreting cells among CD69+, CD8+ T cells from non carriers (n = 9) and carriers (n = 10) of the CCR5Δ32 polymorphism are shown. Horizontal lines represent the median percentage.

To elucidate the functional phenotype of the CD4+ and CD8+ T cells responding to stimulation, activated T cells were gated and evaluated for expression of the activation marker CD69 versus intracellular expression of the cytokines IFN-γ, IL-17 and IL-4. The results are shown in Figure 2 and 3. The percentages IL-4 secreting CD4+ and CD8+ T cells from patients with the CCR5Δ32 genotype was significantly (p = 0.02, respectively p = 0.02) increased compared to patients not carrying the CCR5Δ32 genotype, indicating a more Th2 type directed response. The percentages IFN-γ secreting CD4+ and CD8+ T cells did not significantly differ between carriers and non carriers of CCR5Δ32, meaning the Th1 and Th17 response did not differ between these 2 groups. Comparing the CCR5Δ32 homozygous and heterozygous CD4+ and CD8+ T cells showed no significant differences.

### Frequencies of regulatory T cells

**Figure 4:**
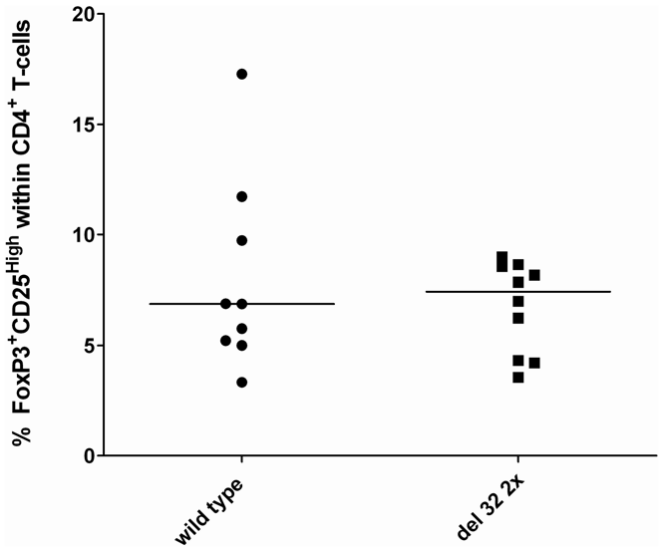
Percentages of Tregs (FoxP3^+^CD25^High^ CD4^+^ T cells) in carriers and non carriers of the CCR5Δ32 polymorphism. Horizontal lines represent the median percentage.

To address the question whether differences in Tregs frequencies influence the distribution of T cell subsets between CCR5Δ32 carriers and non carriers, FoxP3+CD25^High^CD4^+^ T cells were analyzed in both groups. No significant differences were found between the 2 genotype groups (Figure 4). It seems, therefore, that Tregs are not responsible for the differences in Th2 response between CCR5Δ32 carriers and non carriers.

## Discussion

In the present study we demonstrate a skewing of circulating CD4+ and CD8+ T cells towards the Th2 phenotype based on their intracellular cytokine profile after stimulation in ESRD patients carrying the CCR5Δ32 genotype. These data are in line with animal data showing that genetic deficiency of CCR5 results in a shift in immune response towards a Th2 type response, and support the assumption that genetic differences in immune response are involved in the protection against inflammation-associated mortality in ESRD patients reported previously [4].

In ESRD patients cardiovascular disease is a main cause of premature deaths [19]. Chronic inflammation is a major contributing factor [20], [21]. The inflammatory nature of the process of atherosclerosis is nowadays well recognized [22]. In this process T cells play an important role [9]–[12]. Th1 cells, which produce IFN-γ as the principal cytokine, are thought to be pro-inflammatory and pro-atherogenic and are the most prevalent subtype in atherosclerotic lesions; Th2 cells, with IL-4 as the major cytokine, have the ability to inhibit Th1 differentiation and could therefore be anti-atherogenic. The role of Th17 cells, producing IL-17, in atherosclerosis is not yet clear [23]. So, until now atherosclerotic inflammation is regarded as a Th1 directed Th1/Th2 disequilibrium [12].

To our knowledge, this is the first functional study investigating the Th1/Th2 directed immune response in relation to the CCR5Δ32 genetic variant in human. Animal studies consistently show a more pronounced Th2 immune response during genetic deficiency of CCR5 or pharmacological CCR5 blockade in atherosclerotic and other inflammatory conditions. In diet induced atherosclerotic inflammation in mice, genetic deletion of CCR5 was associated with a more stable plaque phenotype and reduced Th1 type immune response of stimulated splenocytes and an increased Th2 type response in splenocytes and lymph node cells [14]. After wire injury in mice with CCR5 deficiency a more atheroprotective immune response was seen, i.e. low IFN-γ and elevated IL-10 in CD4+ splenocytes compaired to CCR5 wild type mice [15]. Also in genetically CCR5 deficient mice with diet induced atherosclerosis, reduced lesion size, increased IL-10 and decreased TNF-α production by CD4+ and CD8+ T cells [16], and reduced macrophage accumulation in plaques and lowered circulating IL-6 levels was seen [17]. In CCR5 deficient mice a more CD4+ Th2 cell activation pattern was seen in colitis in contrast to CCR5 wild type mice [24]. Interestingly, in CCR5 genetically deficient mice who received a renal allograft less Th1 associated markers and increased Th2 associated markers were found during chronic intragraft immune response [25]. In an islet transplantation model it was shown that in genetically CCR5 deficient mice not only in the intragraft immune response but also in the periphery a Th2 shift occurred [26]. In mice with diet-induced atherosclerosis treatment with a RANTES chemokine antagonist, hereby blocking CCR5, reduced atherosclerotic plaque formation, associated with reduced proliferation and secretion of Th1 cytokines IFN-γ and TNF-α, without difference in Th2 cytokine profile [27]. In rats pharmacological CCR5 blockade in stimulated endothelial cells inhibited selective transmigration of CD4+ Th1 cells [28].

Our results extend these animal data on functional differences in immune response for the first time to a human setting and support our previous human cross-sectional association study, showing absence of association between serum CRP and TNF-α levels in ESRD patients carrying the CCR5Δ32 genotype, in contrast to patients without this genetic variant, supporting a reduced Th1 immune response in CCR5Δ32 [29]. It should be emphasized that ELISA and flowcytometry methods give different types of results and that measuring the intracellular cytokine production by FACS is more accurate than ELISA. The measured cytokines by ELISA can be released from several cells, whereas FACS-method identifies the intracellular cytokines produced on a single-cell level. In addition, difference in T cell numbers between the study samples may influence the results obtained from ELISA but not from FACS method hereby probably explaining why we did not find a difference in extracellular cytokine levels between CCR5Δ32 carriers and non carriers. Since Tregs are responsible for regulation and suppression of T cell responses [9], [12], one may argue that differences in Tregs could underlie the increase in IL-4 expression in CCR5Δ32 carriers. However, no significant differences were observed in the percentages of Tregs between CCR5Δ32 carriers and non carriers. Thus, the increased Type 2 response in CCR5Δ32 carriers cannot be related to different frequencies of Tregs.

Together, these findings provide an explanation for the previously observed protection from inflammation-related mortality in ESRD in CCR5Δ32 carriers, as they support a less pro-inflammatory, pro-atherogenic immune response in carriers of the deletion [4]. Our results could also provide a mechanism underlying the protection against atherosclerosis by pharmacological blockade of the CCR5 pathway in animal studies [30]–[32]. Of note, CCR5 blockade has become feasible in humans, and is currently used for treatment of HIV-infection [33]. It has been proposed that CCR5 blockade may be a strategy for protection against inflammation driven cardiovascular disease in ESRD and/or transplantation [34], [35]. Our current results contribute to understanding of mechanisms that could be affected by CCR5 blocking agents in ESRD.

### Limitations

Acknowledged limitation in this study is the relatively small sample size. Besides this one blood sample failed to be stimulated. However, as mentioned above, the results are in accordance with animal data and extend the findings of a correlation study in ESRD patients, supporting the robustness of our findings. Another limitation is that we studied ESRD patients only. ESRD as such affects T-cell properties [36]. Whereas the effects of CCR5 deficiency appear to be remarkably consistent across different species and inflammatory conditions, nevertheless generalization of our results to other populations would require a separate study.

In conclusion, we present the first human data on a difference in Th1/Th2 balance dependent on the CCR5Δ32 genotype in ESRD patients. Stimulated CD4+ and CD8+ T cells of patients with one or two CCR5Δ32 alleles show an increased Th2 type phenotype base on their intracellular cytokine profile. Differences in immune response may be involved in the impact of CCR5Δ32 on outcome in ESRD, and possible other inflammatory conditions.
